# Supplementary material for: Maternal RNA transcription in Dlk1-Dio3 domain is critical for proper development of the mouse placental vasculature
Source: Commun Biol. 2024 Mar 23;7:363. doi: 10.1038/s42003-024-06038-3 (PMC10960817; doi:10.1038/s42003-024-06038-3)
Supplement: Supplementary file 2 — Supplementary Information [file 42003_2024_6038_MOESM2_ESM.pdf]

**Supplementary Information**

# Maternal RNA transcription in *Dlk1-Dio3* domain is critical for proper development of the mouse placental vasculature

Ximeijia Zhang[1], Hongjuan He[1], Haoran Yu[1], Xiangqi Teng[1], Ziwen Wang[1], Chenghao Li[1], JiaHang Li[1], Haopeng Yang[1], Jiwei Shen[1], Tong Wu[1], Fengwei Zhang[1], Yan Zhang[1], Qiong Wu[1, ✉]

[1]School of Life Science and Technology, State Key Laboratory of Urban Water Resource and Environment, Harbin Institute of Technology, Harbin 150006, Heilongjiang, China
✉e-mail: kigo@hit.edu.cn

## Supplementary Figures

**Supplementary Figure 1** Gross phenotypes of *Gtl2* polyA knock-in yolk sac.

**Supplementary Figure 2** *Gtl2* polyA knock-in doesn't alter the morphology of maternal decidua and junctional zone.

**Supplementary Figure 3** The labyrinth of *Gtl2* polyA knock-in placentas at different embryonic days.

**Supplementary Figure 4** Verification of some DEGs with significant changes in RNA-seq by qRT-PCR using independent biological replicates.

**Supplementary Figure 5** The KEGG and GO enrichment analysis of upregulated DEGs in *Gtl2* polyA knock-in placentas.

**Supplementary Figure 6** The KEGG and GO enrichment analysis of downregulated DEGs in *Gtl2* polyA knock-in placentas.

**Supplementary Figure 7** The GO enrichment terms of DEGs are significant in MKI and HOMO, and not significant in PKI.

**Supplementary Figure 8** The results of strand-specific RT-PCR of *Dlk1* and the results of IG-DMR, *Gtl2*-DMR and *Dlk1*-DMR in the other two biological repliacates.

## Supplementary Tables

**Supplementary Table 1** Numbers of *Gtl2* polyA knock-in placentas and survival rates at different stages.

**Supplementary Table 2** The sequences of sgRNA and ssDNA donor.

**Supplementary Table 3** Primer sequences.

## Supplementary Data

**Supplementary Data 1** All source data for graphs in the figures.

**Supplementary Figures**

**Supplementary Fig1**

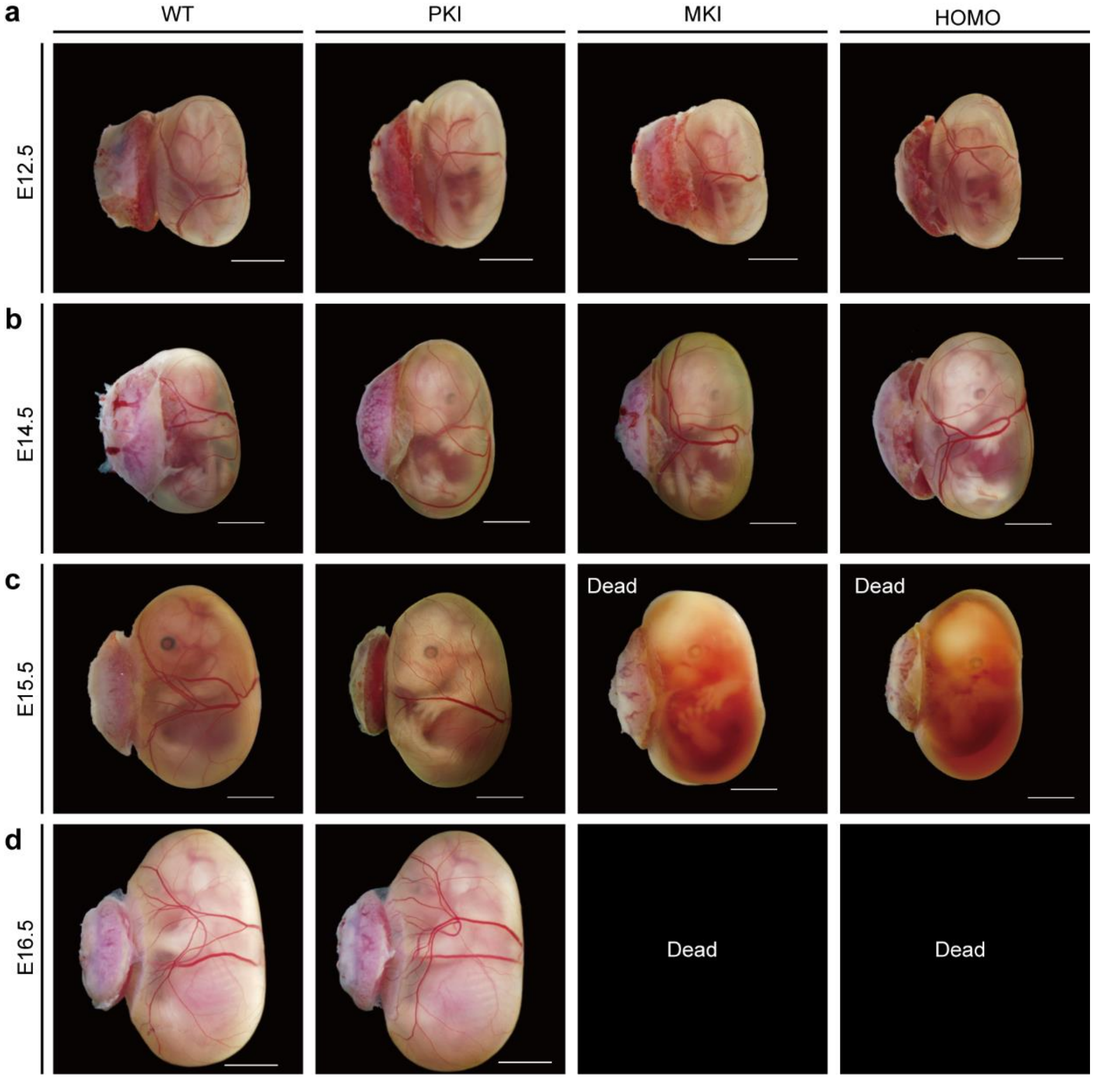


**Supplementary Figure 1** Gross phenotypes of *Gtl2* polyA knock-in yolk sac.

(a-d) Gross phenotypes of WT, PKI, MKI and HOMO yolk sac at E12.5, E14.5, E15.5 and E16.5. Scale bars: 2mm.

**Supplementary Fig2**

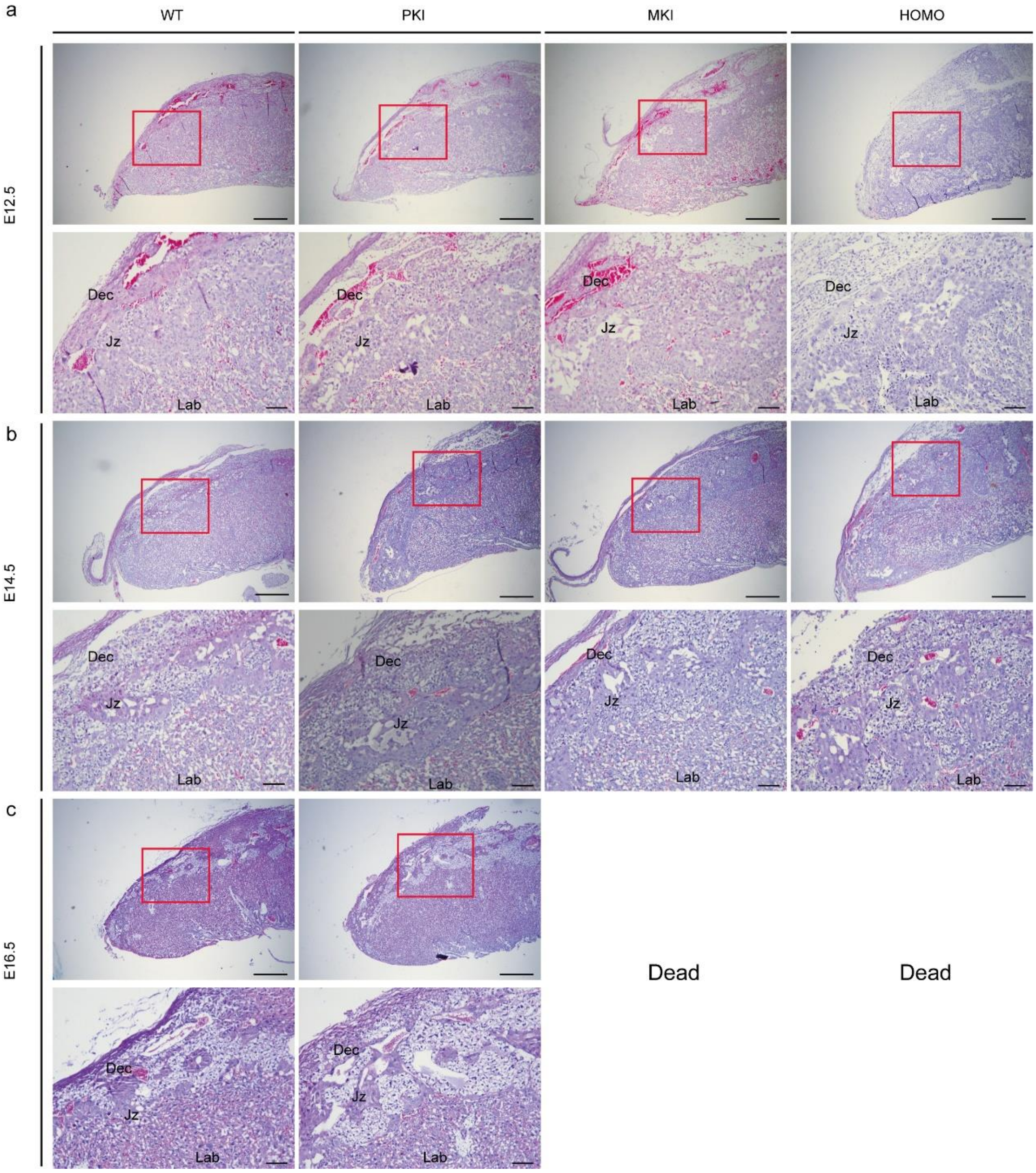


**Supplementary Figure 2** *Gtl2* polyA knock-in doesn’t alter the morphology of maternal decidua and junctional zone.

(a-c) H&E staining of the placentas of the survival embryos at E12.5, E14.5 and E16.5. Red boxes show high-magnification images of maternal decidua and junctional zone. Dec: decidua, Jz: junctional zone, Lab: labyrinth. Scale bars of the first, third and fifth rows: 500μm, scale bars of the second, fourth and sixth rows: 100μm.

**Supplementary Fig3**

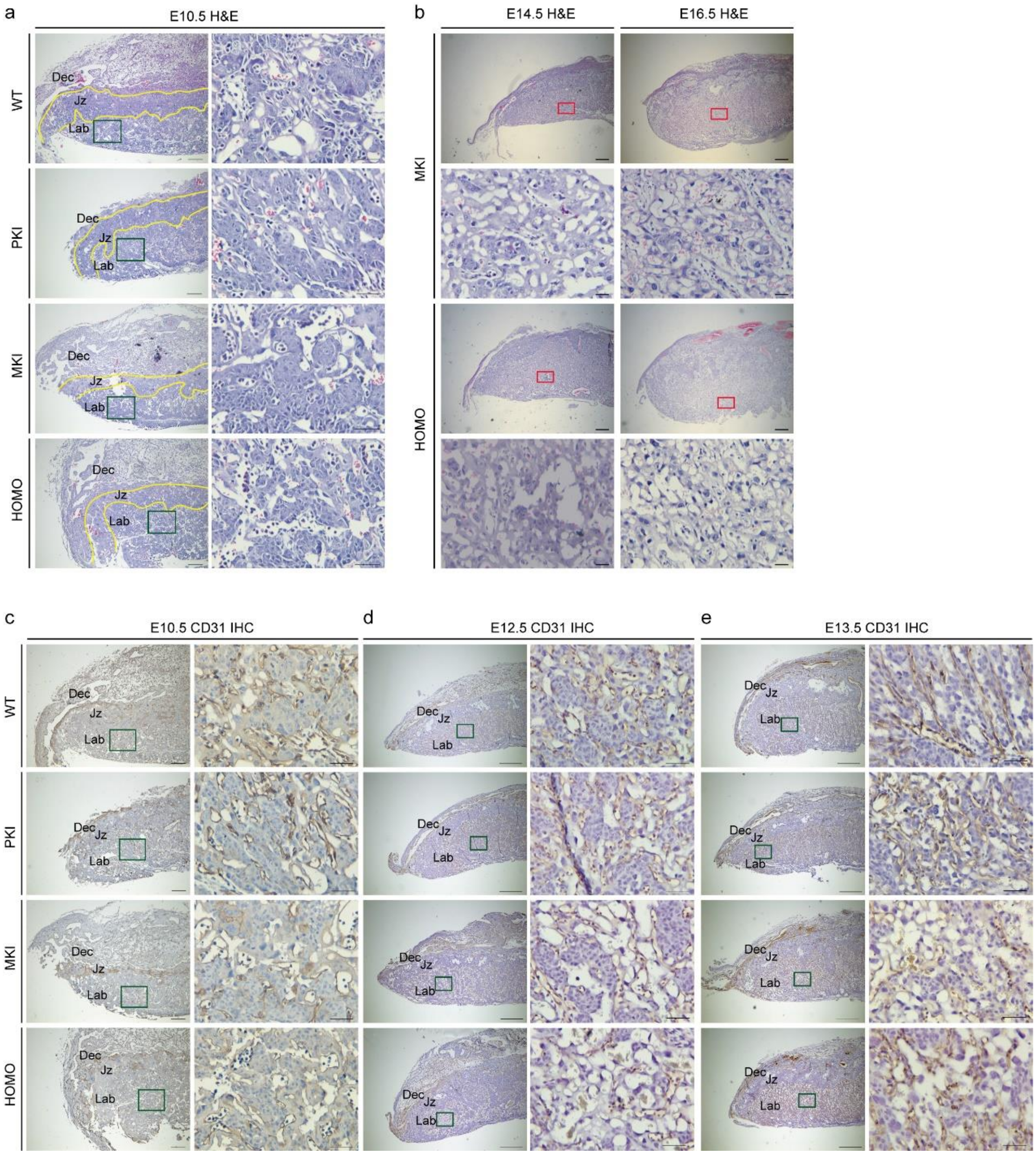


**Supplementary Figure 3** The labyrinth of *Gtl2* polyA knock-in placentas at different embryonic days.

(a) H&E staining of *Gtl2* polyA knock-in placentas at E10.5. Dark green boxes show high-magnification images of labyrinth areas. Scale bars of the first column: 200μm, scale bars of the second column: 50μm. (b) H&E staining of MKI and HOMO placentas of the dead embryos at E14.5 and E16.5. Red boxes show high-magnification images of labyrinth areas. Scale bars of the first and third rows: 500μm, scale bars of the second and fourth rows: 50μm. (c) (d) (e) CD31 IHC of *Gtl2* polyA knock-in placentas at E10.5, E12.5, E13.5. Dark green boxes show high-magnification images of labyrinth areas. Scale bars of the first, third and fifth columns: 200μm, scale bars of the second fourth, and sixth columns: 50μm.

**Supplementary Fig4**

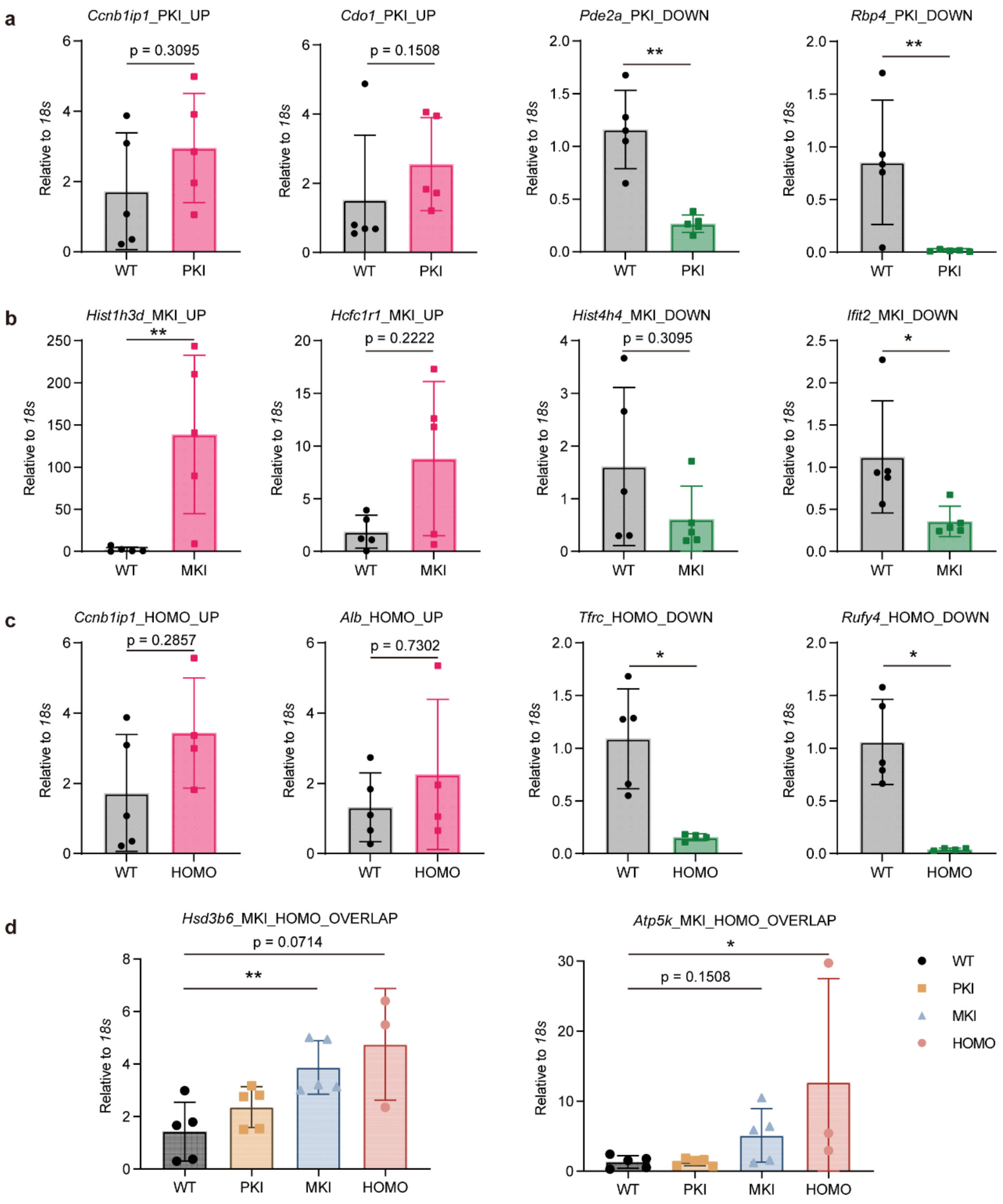


**Supplementary Figure 4** Verification of some DEGs with significant changes in RNA-seq by qRT-PCR using independent biological replicates.

(a) (b) (c) qRT-PCR confirm the expression of two upregulated and two downregulated DEGs in PKI, MKI and HOMO placentas, respectively. In (a) (b) (c), biological duplication: WT n=5, PKI n=5, MKI n=5, HOMO n=4. The mean expression ± SD of each genotype is plotted. Student t-test is used to analyze the p values. *p<0.05, **p<0.01. (d) qRT-PCR confirm the expression of two DEGs upregulated in MKI and HOMO, but not upregulated in PKI. In (d), biological duplication: WT n=5, PKI n=5, MKI n=5, HOMO n=3. The mean expression ± SD of each genotype is plotted. Student t-test is used to analyze the p values. *p<0.05, **p<0.01.

## Supplementary Fig5

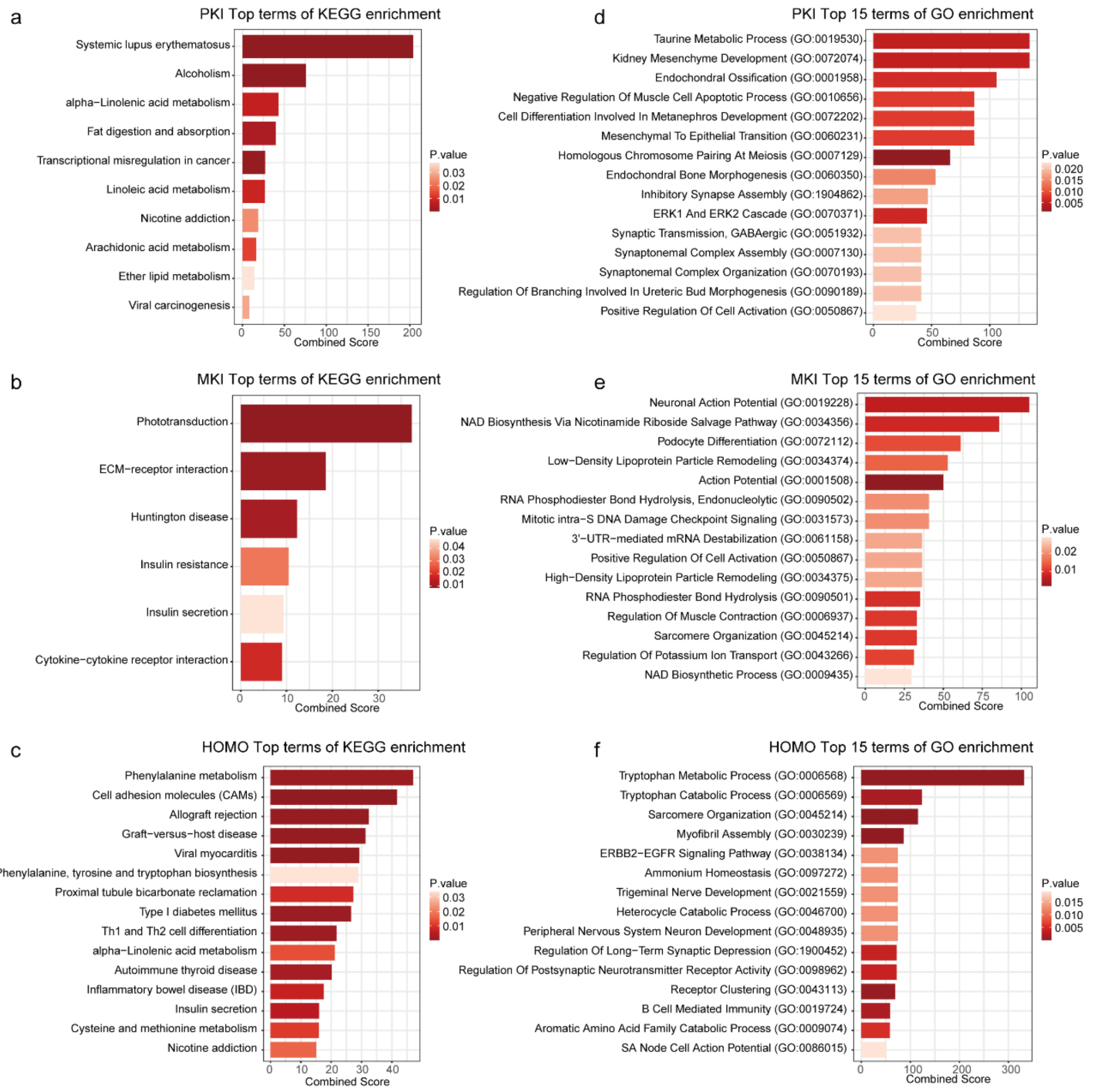


**Supplementary Figure 5** The KEGG and GO enrichment analysis of upregulated DEGs in *Gtl2* polyA knock-in placentas.

(a) (b) (c) The top KEGG enrichment terms of upregulated DEGs in PKI, MKI and HOMO placentas, respectively. (d) (e) (f) The top 15 GO enrichment terms of upregulated DEGs in PKI, MKI and HOMO placentas, respectively.

## Supplementary Fig6

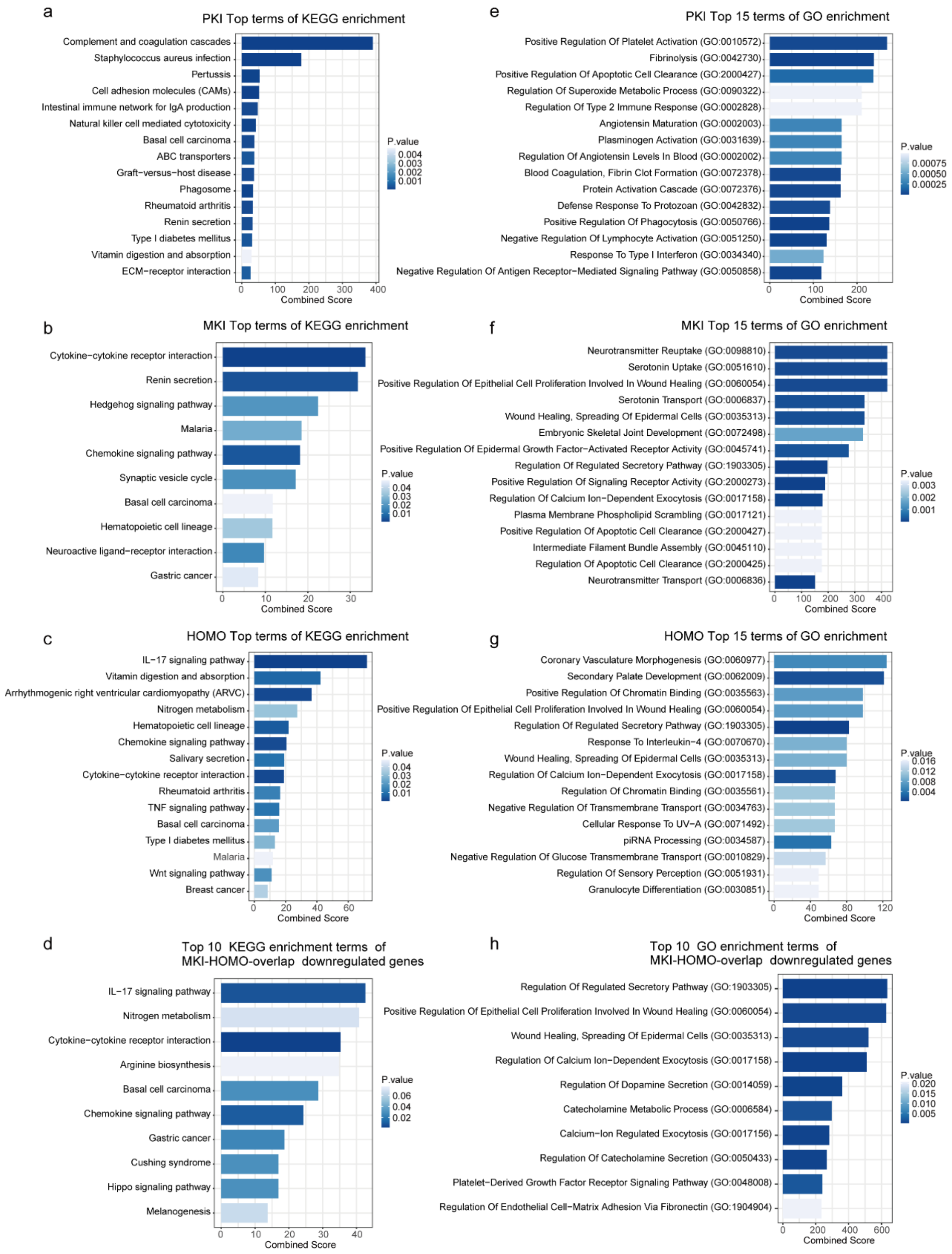


**Supplementary Figure 6** The KEGG and GO enrichment analysis of downregulated DEGs in *Gtl2* polyA knock-in placentas.

(a) (b) (c) (d) The top KEGG enrichment terms of PKI downregulated DEGs, MKI downregulated DEGs, HOMO downregulated DEGs and MKI-HOMO overlapped downregulated DEGs, respectively. (e) (f) (g) (h) The top GO enrichment terms of PKI downregulated DEGs, MKI downregulated DEGs, HOMO downregulated DEGs and MKI-HOMO overlapped downregulated DEGs, respectively.

**Supplementary Fig7**

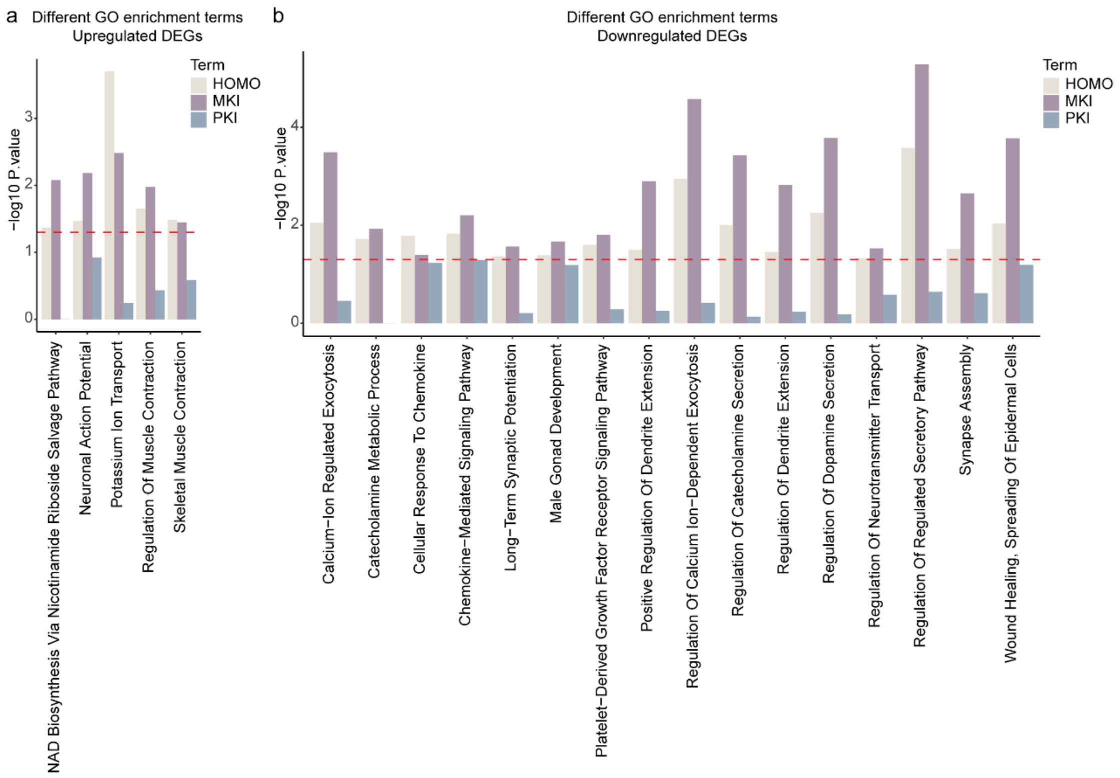


**Supplementary Figure 7** The GO enrichment terms of DEGs are significant in MKI and HOMO, and not significant in PKI.

(a) The GO enrichment terms of upregulated DEGs are significant in MKI and HOMO, and not significant in PKI. (b) The GO enrichment terms of downregulated DEGs are significant in MKI and HOMO, and not significant in PKI. The red dashed line represents the p is 0.05.

## Supplementary Fig8

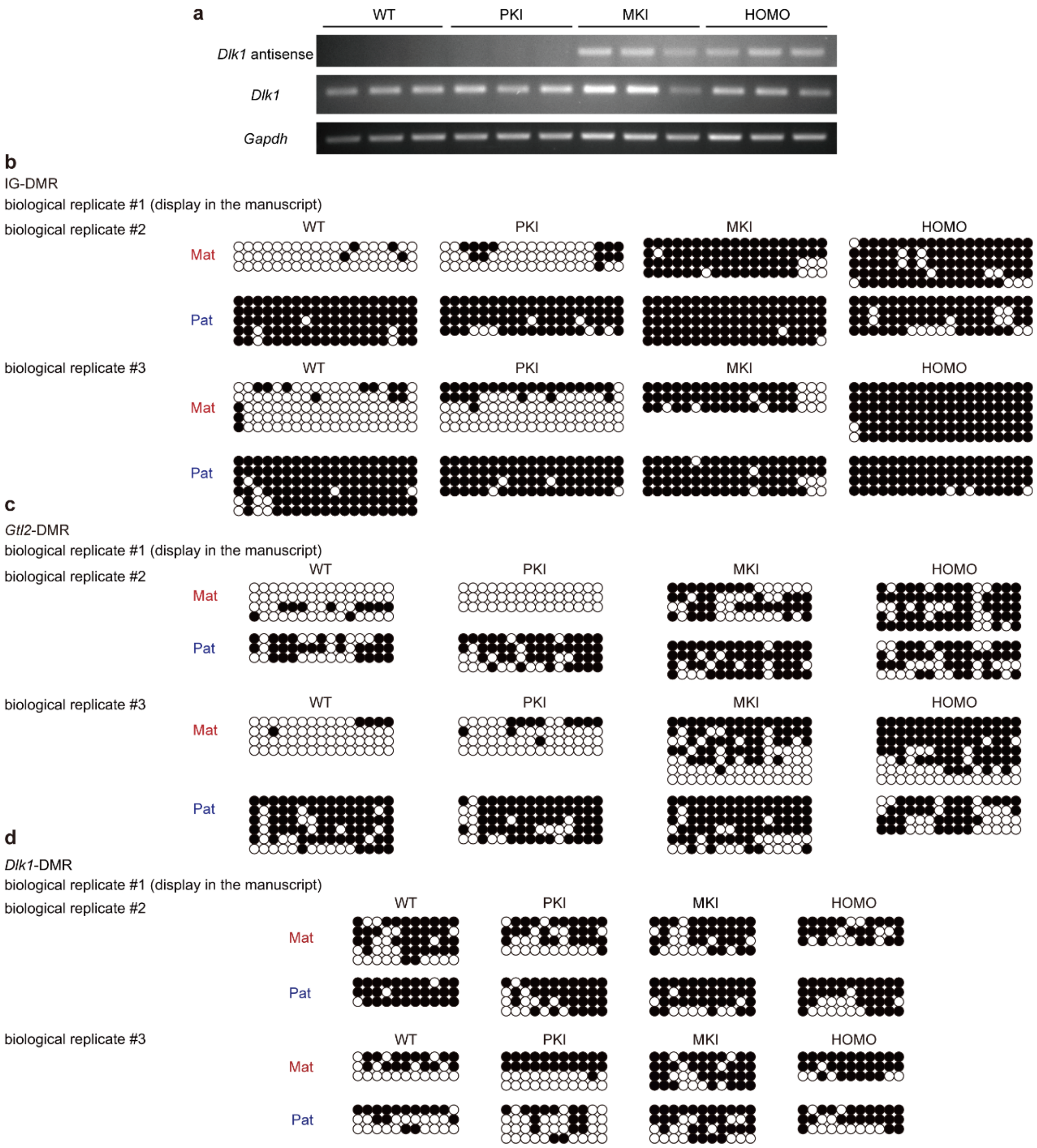


**Supplementary Figure 8** The results of strand-specific RT-PCR of *Dlk1* and the results of IG-DMR, *Gtl2*-DMR and *Dlk1*-DMR in the other two biological repliacates.

(a) Strand-specific RT-PCR detected that activation of *Dlk1* expression came from sense strand, not in antisense strand in WT and PKI placentas. Activation of *Dlk1* expression came from both sense strand and antisense strand in MKI and HOMO placentas, and *Dlk1* had a similar expression level in both stands. (b) (c) (d) Methylation status of IG-DMR, *Gtl2*-DMR and *Dlk1*-DMR in *Gtl2* polyA knock-in and WT placentas separately. Each row represents one clone. SNPs are used to distinguish paternal allele clones from maternal allele clones. Filled and hollow circles indicate methylated and unmethylated CpG sites, respectively.

## Supplementary Tables

**Supplementary Table 1** Numbers of *Gtl2* polyA knock-in placentas and survival rates at different stages.

| Embryonic day | Population size /The number of the dead | | | | Survival rate | | | |
|---|---|---|---|---|---|---|---|---|
| | WT | PKI | MKI | HOMO | WT | PKI | MKI | HOMO |
| E12.5 | 52/0 | 32/0 | 35/0 | 13/0 | 100% | 100% | 100% | 100% |
| E13.5 | 14/0 | 15/0 | 12/2 | 3/3 | 100% | 100% | 83.33% | 0% |
| E14.5 | 78/0 | 42/0 | 35/16 | 37/18 | 100% | 100% | 54.29% | 51.35% |
| E15.5 | 4/0 | 6/0 | 15/12 | 5/5 | 100% | 100% | 20% | 0% |
| E16.5 | 17/0 | 11/0 | 2/2 | 2/2 | 100% | 100% | 0% | 0% |

**Supplementary Table 2** The sequences of sgRNA and ssDNA donor.

| **Name** | **Sequence** |
|---|---|
| sgRNA | GCAGCTCTTCCAGAAACC |
| ssDNA donor | ATATAAACCCCACCCAGCCAGCCCCTAGCACAGAAGACGAAGA<br>GCTGGAATAGAGCTCGCCTCGGCTCTGCTGGCCTTGGCTGCAG<br>CTCTTCCAGAAAAATAAAAGATCTTTATTTTCATTAGATCTGTGT<br>GTTGGTTTTTTGTGTGAATAAAAGATCTTTATTTTCATTAGATCT<br>GTGTGTTGGTTTTTTGTGTGAATAAAAGATCTTTATTTTCATTAG<br>ATCTGTGTGTTGGTTTTTTGTGTGCCCGGGGCGCCCACAGAAGA<br>ATCTCTTACCTGGTGAGTGGTTAGCCATCCTTTGCCTGAAAGGA<br>TGTGCAAAAATGAAGACGACATCACTATCTGG |

**Supplementary Table 3** Primer sequences.

| **Primer name** | **Primer sequence (5' to 3')** | **Purpose** |
|---|---|---|
| Genotyping-F | AATGCGCTGACGTCAAAGACCA | Primers for genotyping |
| Genotyping-R | GGCTTTCAGCCACCAAAACCAG | |
| 18s-F | CTCATGTGGTGTTGAGGAAAGCAG | Primers for qRT-PCR |
| 18s-R | TGTTGTCTAGACCGTTGGCCAGAA | |
| Dlk1-F | ACGGGAAATTCTGCGAAATA | |
| Dlk1-R | CTTTCCAGAGAACCCAGGTG | |
| Rtl1-F | CCGAGGGCTCATCCAACACCGTTGA | |
| Rtl1-R | CCTGGGCTGGGCCACTATCTGA | |
| Dio3-F | GGAGAAGGGGAAGAACTTGG | |
| Dio3-R | TCAGAGCAACTTCCTTCAAGTC | |
| Gtl2-F | CGAGGACTTCACGCACAAC | |
| Gtl2-R | TTACAGTTGGAGGGTCCTGG | |
| Rian-F | TAGAGTCTCCCTTGAAAGTGG | |
| Rian-R | TGGTATCTATAAGAACAGAGCTGA | |
| Mirg-F | GTTGTCTGTGATGAGTTCGC | |
| Mirg-R | CCTTGAACATCCGCTCC | |
| miR-337-5p-F | CGGCGTCATGCAGGAGTTGATT | |
| miR-337-5p-R | mRQ 3’ Primer From Takara | |
| miR-127-3p-F | TCGGATCCGTCTGAGCTTGGCT | |
| miR-127-3p-R | mRQ 3’ Primer From Takara | |
| miR-370-3p-F | GCCTGTTGGACCCAGGAAGGT | |
| miR-370-3p-R | AGCACCCGAGCGATGGTGA | |
| miR-541-5p-F | AAGGGATTCTGATGTTGGTCACACT | |
| miR-541-5p-R | mRQ 3’ Primer From Takara | |
| miR-381-3p-F | TATACAAGGGCAAGCTCTCTGT | |
| miR-381-3p-R | mRQ 3’ Primer From Takara | |
| Ccnb1ip1-qrt-F | GGTACACCAGGAGCGTCTCTA | |
| Ccnb1ip1-qrt-R | GCTTTTGGTACTGGCGATTACG | |
| Cdo1-qrt-F | GGGGACGAAGTCAACGTGG | |
| Cdo1-qrt-R | ACCCCAGCACAGAATCATCAG | |
| Pde2a-qrt-F | TGGCGTTGTGGACGATGAG | |
| Pde2a-qrt-R | CGCGATAGAAAAGCGGATGG | |
| Rbp4-qrt-F | AGTCAAGGAGAACTTCGACAAGG | |
| Rbp4-qrt-R | CTTGGCTGTGGCGCTCATA | |
| Hist1h3d-qrt-F | ACCGTTCTCATTCCCTGAGAC | |
| Hist1h3d-qrt-R | GTAGCGGTGAGGCTTCTTCA | |
| Hcfc1r1-qrt-F | GGGTAACTCGCGGCCTAAAC | |
| Hcfc1r1-qrt-R | AGGGCTTCGGGAAAAGTCAC | |
| Hist4h4-qrt-F | GAAGCGCATCTCGGGTCTC | |
| Hist4h4-qrt-R | CATAGCCGTAACCGTCTTGC | |
| Ifit2-qrt-F | AGTACAACGAGTAAGGAGTCACT | |

| | | |
|---|---|---|
| Ifit2-qrt-R | AGGCCAGTATGTTGCACATGG | |
| Alb-qrt-F | TGCTTTTTCCAGGGGTGTGTT | |
| Alb-qrt-R | TTACTTCCTGCACTAATTTGGCA | |
| Tfrc-qrt-F | GTTTCTGCCAGCCCCTTATTAT | |
| Tfrc-qrt-R | GCAAGGAAAGGATATGCAGCA | |
| Rufy4-qrt-F | GGAGCAGGGCTAAGGATGTTA | |
| Rufy4-qrt-R | TCAGGCTGATTCCACTCACAC | |
| Hsd3b6-qrt-F | TGATGGGAAGAGGGTGGAG | |
| Hsd3b6-qrt-R | AGGTGCTGAGAGGCTTGGA | |
| Atp5k-qrt-F | TCATCGGCATGGCATACGG | |
| Atp5k-qrt-R | CCGCTGCTATTCTCCTCTCCT | |
| Dlk1-imprint-F | TCCTGAAGGTGTCCATGAAAGAGC | Primers for biallelic expression |
| Dlk1-imprint-R | AAGCATAGCGTTCACTCGATTCCAC | |
| Dlk1-ISH-F | CCTCTTGCTCCTGCTGGCTTTC | Primers for ISH probes |
| Dlk1-ISH-R | GATGTGTTGCTCGGGCTGCTG | |
| Gtl2-ISH-F | GGGAAATTGGAGGTGAGG | |
| Gtl2-ISH-R | GGACAAGCGACAAAGAGG | |
| miR-127-ISH-F | GCTCCATGTAGCTGGTCGAA | |
| miR-127-ISH-R | GGCATGAAGTGGCTCCGAA | |
| Rtl1-ISH-F | GCTCCATGTAGCTGGTCGAA | |
| Rtl1-ISH-R | GGCATGAAGTGGCTCCGAA | |
| Rian-ISH-F | CAAATCTCCATGCACGGAAAG | |
| Rian-ISH-R | GCCTTGACCATCATGAAGAC | |
| Tpbpa-ISH-F | TCCAAGGACCTCTGAAGAGC | |
| Tpbpa-ISH-R | AGGATCCCACTTGTCAGGGG | |
| IG-DMR-F | GTATTGTAATATAGGTTAGGTG | Primers for DNA methylation |
| IG-DMR-R1 | CTACATAATACCATATAAACATATCTC | |
| IG-DMR-R2 | CACAACTACACAAAATACTAC | |
| Gtl2-DMR-F | AAATTTTGTAAGGAAAAGAATTTTTAGG | |
| Gtl2-DMR-R | TTCAAAATTACTAATCAACATAAACCTC | |
| Dlk1-DMR-F | TTTTGAAGGTGTTTATGAAAGAGTTTAA | |
| Dlk1-DMR-R | CATCACCAACCTCCTTATTAAAAATAA | |
