# Supplementary material for: Maternal RNA transcription in Dlk1-Dio3 domain is critical for proper development of the mouse placental vasculature
Source: Commun Biol. 2024 Mar 23;7:363. doi: 10.1038/s42003-024-06038-3 (PMC10960817; doi:10.1038/s42003-024-06038-3)
Supplement: Supplementary file 5 — Reporting Summary [file 42003_2024_6038_MOESM5_ESM.pdf]

nature portfolio

Corresponding author(s): Qiong Wu

Last updated by author(s): 2024/02/04

Reporting Summary

Nature Portfolio wishes to improve the reproducibility of the work that we publish. This form provides structure for consistency and transparency in reporting. For further information on Nature Portfolio policies, see our Editorial Policies and the Editorial Policy Checklist.

Please do not complete any field with "not applicable" or n/a. Refer to the help text for what text to use if an item is not relevant to your study.
For final submission: please carefully check your responses for accuracy; you will not be able to make changes later.

Statistics

For all statistical analyses, confirm that the following items are present in the figure legend, table legend, main text, or Methods section.

n/a	Confirmed	
☐	☒	The exact sample size (*n*) for each experimental group/condition, given as a discrete number and unit of measurement
☐	☒	A statement on whether measurements were taken from distinct samples or whether the same sample was measured repeatedly
☐	☒	The statistical test(s) used AND whether they are one- or two-sided<br>*Only common tests should be described solely by name; describe more complex techniques in the Methods section.*
☐	☒	A description of all covariates tested
☐	☒	A description of any assumptions or corrections, such as tests of normality and adjustment for multiple comparisons
☐	☒	A full description of the statistical parameters including central tendency (e.g. means) or other basic estimates (e.g. regression coefficient) AND variation (e.g. standard deviation) or associated estimates of uncertainty (e.g. confidence intervals)
☐	☒	For null hypothesis testing, the test statistic (e.g. *F*, *t*, *r*) with confidence intervals, effect sizes, degrees of freedom and *P* value noted<br>*Give P values as exact values whenever suitable.*
☒	☐	For Bayesian analysis, information on the choice of priors and Markov chain Monte Carlo settings
☐	☒	For hierarchical and complex designs, identification of the appropriate level for tests and full reporting of outcomes
☒	☐	Estimates of effect sizes (e.g. Cohen's *d*, Pearson's *r*), indicating how they were calculated

Our web collection on statistics for biologists contains articles on many of the points above.

Software and code

Policy information about availability of computer code

Data collection	All slides were imaged using software ZEN (ZEISS). ImageJ was used for area statistics.
Data analysis	Statistical analyses were performed using GraphPad Prism 9. Cytoscape was used to analyze gene degrees. Chromas was used to analyze biallelic expression. DNAMAN 9 was used to do methylation analysis. R packages (HISAT2, StringTie, EdgeR, ClusterProfiler) were used to do RNA-seq analysis.

For manuscripts utilizing custom algorithms or software that are central to the research but not yet described in published literature, software must be made available to editors and reviewers. We strongly encourage code deposition in a community repository (e.g. GitHub). See the Nature Portfolio guidelines for submitting code & software for further information.

Data

Policy information about availability of data

All manuscripts must include a data availability statement. This statement should provide the following information, where applicable:

- Accession codes, unique identifiers, or web links for publicly available datasets
- A description of any restrictions on data availability
- For clinical datasets or third party data, please ensure that the statement adheres to our policy

RNA-seq data have been deposited in Gene Expression Omnibus database under accession number GSE255900. All the other data generated in this study are included in the article and the supplementary information.

Research involving human participants, their data, or biological material

Policy information about studies with human participants or human data. See also policy information about sex, gender (identity/presentation), and sexual orientation and race, ethnicity and racism.

Reporting on sex and gender	No human participants was involved in this study.
Reporting on race, ethnicity, or other socially relevant groupings	N/A
Population characteristics	N/A
Recruitment	N/A
Ethics oversight	N/A

Note that full information on the approval of the study protocol must also be provided in the manuscript.

Field-specific reporting

Please select the one below that is the best fit for your research. If you are not sure, read the appropriate sections before making your selection.

☒ Life sciences ☐ Behavioural & social sciences ☐ Ecological, evolutionary & environmental sciences

For a reference copy of the document with all sections, see nature.com/documents/nr-reporting-summary-flat.pdf

Life sciences study design

All studies must disclose on these points even when the disclosure is negative.

Sample size	Sample size was determined based on previous publications using similar paradigms, and sufficient for data analysis .
Data exclusions	No data was excluded for analysis in this study.
Replication	Data can be reproduced.
Randomization	The mice were randomly selected according to WT, PKI, MKI and HOMO genotypes.
Blinding	The investigators were blinded to group allocation during data collection.

Reporting for specific materials, systems and methods

We require information from authors about some types of materials, experimental systems and methods used in many studies. Here, indicate whether each material, system or method listed is relevant to your study. If you are not sure if a list item applies to your research, read the appropriate section before selecting a response.

Materials & experimental systems

n/a	Involved in the study	
☐	☒	Antibodies
☒	☐	Eukaryotic cell lines
☒	☐	Palaeontology and archaeology
☐	☒	Animals and other organisms
☒	☐	Clinical data
☒	☐	Dual use research of concern
☒	☐	Plants

Methods

n/a	Involved in the study	
☒	☐	ChIP-seq
☒	☐	Flow cytometry
☒	☐	MRI-based neuroimaging

Antibodies

Antibodies used	rabbit monoclonal anti-CD31 (1:100, #77699, CST),rabbit monoclonal anti-DLK1 (1:200, ab210471, Abcam), HRP Goat Anti-Rabbit IgG (1:5000, AS014, ABclonal)
Validation	Antibodies were validated by manufacturers (CST, Abcam, and ABclonal websites).

Animals and other research organisms

Policy information about studies involving animals; ARRIVE guidelines recommended for reporting animal research, and Sex and Gender in Research

Laboratory animals	DBA/2J and C57BL/6N mice at the age of ~8 weeks.
Wild animals	This study did not involve wild animals .
Reporting on sex	Placentas were collected from four genotypes embryos comprised of both sexes.
Field-collected samples	This study did not involve field-collected samples.
Ethics oversight	All experimental procedures were approved by the Institutional Animal Care and Use Committee (IACUC - 2023066) of Harbin Institute of Technology.

Note that full information on the approval of the study protocol must also be provided in the manuscript.
